# Pulp regeneration by transplantation of dental pulp stem cells in pulpitis: a pilot clinical study

**DOI:** 10.1186/s13287-017-0506-5

**Published:** 2017-03-09

**Authors:** Misako Nakashima, Koichiro Iohara, Masashi Murakami, Hiroshi Nakamura, Yayoi Sato, Yoshiko Ariji, Kenji Matsushita

**Affiliations:** 1 Department of Stem Cell Biology and Regenerative Medicine, National Center for Geriatrics and Gerontology, Obu, Japan; 2 Department of Endodontics, School of Dentistry, Aichi Gakuin University, Nagoya, Japan; 3 Innovation Center for Clinical Research, National Center for Geriatrics and Gerontology, Obu, Japan; 4 Department of Oral and Maxillofacial Radiology, School of Dentistry, Aichi Gakuin University, Nagoya, Japan; 5 Department of Dental and Oral Infrastructure Development, Center of Advanced Medicine for Dental and Oral Diseases, National Center for Geriatrics and Gerontology, Obu, Japan

**Keywords:** Clinical study, Pulp regeneration, Mobilized dental pulp stem cells (Mobilized DPSCs), Autologous cell transplantation, Granulocyte colony-stimulating factor (G-CSF), Pulpectomy, Good manufacturing practice (GMP)

## Abstract

**Background:**

Experiments have previously demonstrated the therapeutic potential of mobilized dental pulp stem cells (MDPSCs) for complete pulp regeneration. The aim of the present pilot clinical study is to assess the safety, potential efficacy, and feasibility of autologous transplantation of MDPSCs in pulpectomized teeth.

**Methods:**

Five patients with irreversible pulpitis were enrolled and monitored for up to 24 weeks following MDPSC transplantation. The MDPSCs were isolated from discarded teeth and expanded based on good manufacturing practice (GMP). The quality of the MDPSCs at passages 9 or 10 was ascertained by karyotype analyses. The MDPSCs were transplanted with granulocyte colony-stimulating factor (G-CSF) in atelocollagen into pulpectomized teeth.

**Results:**

The clinical and laboratory evaluations demonstrated no adverse events or toxicity. The electric pulp test (EPT) of the pulp at 4 weeks demonstrated a robust positive response. The signal intensity of magnetic resonance imaging (MRI) of the regenerated tissue in the root canal after 24 weeks was similar to that of normal dental pulp in the untreated control. Finally, cone beam computed tomography demonstrated functional dentin formation in three of the five patients.

**Conclusions:**

Human MDPSCs are safe and efficacious for complete pulp regeneration in humans in this pilot clinical study.

## Background

Dental caries is a common health problem in humans. When dental caries is deep, reaching the dental pulp, the treatment of choice is generally pulpectomy. The dental pulp has several vital functions such as protection from infections by immunological surveillance, rapid reparative dentin formation to guard against noxious external stimuli, and maintenance of tensile strength to prevent tooth fractures [1]. Following pulpectomy and root canal filling, postoperative pain [2], apical periodontal lesions caused by microleakage from the tooth crown [3, 4], and vertical root fracture [5] may occur, leading to a higher incidence of extraction of the affected tooth. Recent advances in stem cell biology have aided stem cell therapy to regenerate the pulp/dentin complex for conservation and complete structural and functional restoration of the tooth by the triad of tissue engineering: 1) mesenchymal stem cells (MSCs), 2) growth/differentiation factors or cytokines, and migration/homing factors, and 3) the microenvironment (scaffold, extracellular matrix) [6]. We have demonstrated complete pulp regeneration by harnessing autologous dental pulp stem cell (DPSCs) subsets transplanted with stromal cell-derived factor 1 (SDF1) in a collagen scaffold into a canine pulpitis model [7, 8]. Next, a novel isolation method was developed employing an optimal granulocyte colony-stimulating factor (G-CSF)-induced mobilization of DPSCs for clinical-grade mesenchymal stem cells from a small amount of pulp tissue by good manufacturing practice (GMP)-grade guidelines [9]. G-CSF was already approved by the Food and Drug Administration (FDA) for clinical use. The isolated human mobilized DPSCs (MDPSCs) were characterized further by the higher migratory activity and trophic effects including migration, anti-apoptosis, and immunosuppression compared with colony-derived DPSCs in vitro. Furthermore, human MDPSCs demonstrated higher regeneration potential using an ectopic tooth root transplantation in severe combined immunodeficient (SCID) mice. Thus, MDPSCs have potential utility for pulp regeneration [9]. G-CSF was evaluated as an optimal GMP-grade migration/homing factor for pulp regeneration, having a variety of effects including anti-apoptosis on the transplanted and migrated cells, engraftment of the transplanted cells, angiogenesis, and immunosuppression [10]. The potential stem cell therapy for pulpitis harnessing MDPSCs with G-CSF was then examined in a preclinical study. Initially, the human MDPSCs isolated in a totally enclosed system in a GMP-compliant facility were evaluated by their karyotype, safety, and efficacy. Then, canine MDPSCs were isolated by the similar standard operating procedure (SOP) used in humans, and the preclinical feasibility, safety, and efficacy of pulp regeneration was established by autologous transplantation of the MDPSCs with GMP-grade G-CSF into the pulpectomized tooth in a canine pulpitis model [10]. On the basis of these preclinical safety and efficacy results and its mechanism for pulp regeneration, the protocol of a clinical study for pulp regenerative therapy was developed and approved by Institutional Review Boards and by the Japanese Ministry of Health, Labor and Welfare.

The aim of this investigation is to assess the safety, potential efficacy, and feasibility of autologous transplantation of human clinical-grade MDPSCs and to evaluate the utility of the stem cell therapy in a pilot clinical study for the first time. According to the Japanese guidelines of human stem cell clinical research, based on ethical considerations, only cases in which pulp tissue removal is inevitable should be selected for clinical study. In cases of severe irreversible pulpitis, including chronic ulcer pulpitis and acute suppurative pulpitis, the pulp tissue is exposed and the whole pulp tissue is infected, and there is no effective treatment other than whole pulp removal. Thus, we selected pulpectomized teeth due to severe irreversible pulpitis without periapical lesions for this purpose.

## Methods

### Patients

The pilot clinical study was conducted according to the principles of the Declaration of Helsinki and the Japanese guidelines of human stem cell clinical research, and to the standard of the manufacturing management and the quality control of pharmaceutical products and quasi-drug (Good Manufacturing Practice; GMP). Subjects were enrolled if they fulfilled the following inclusion criteria: aged between 20 and 55 years, diagnosis of irreversible pulpitis of single root canal, no fracture, a sound tooth structure remaining over the margin of the alveolar bone and no periapical radiolucency by X-ray analysis, and having a discarded tooth without deep caries to supply pulp tissue. Patients were excluded if they presented evidence of infection due to virus, bacteria, fungi and mycoplasma, severe cardiovascular disease, diabetes (HbA1c (NGSP) over 7.0%), osteoporosis, pregnancy, were mentally disabled or had mental disease. In addition patients who received antiplatelet agents or anticoagulant remedy and who had a history of allergy to antimicrobial and the local anesthetic agents and positive intracutaneous reaction for atelocollagen were excluded. Patients who could not receive magnetic resonance imaging (MRI) examination were also excluded. The enrolled patients for participation in the clinical study underwent autologous serum isolation and further extraction of a discarded tooth after signing informed consent again.

### Isolation and in vitro expansion of MDPSCs

Autologous serum was isolated from freshly collected blood (200 ml) by Serum Collection Set (CELLAID®, JMS Co. Ltd., Hiroshima, Japan) in a GMP-compliant facility. The autologous discarded tooth was extracted, soaked in Hank’s balanced salt solution (Invitrogen, Carlsbad, CA, USA) after making a longitudinal cut, and transported to the GMP-compliant facility within 1 hour under strict temperature control at 0–10 °C (Testo, Yokohama, Japan). The isolation of MDPSCs was performed according to a standard operating procedure (SOP) under strict GMP conditions in a totally enclosed system of the Isolator (Panasonic Healthcare Co. Ltd., Tokyo, Japan) as described previously in the preclinical trial [10]. In brief, the pulp cells were isolated by enzymatic digestion in 0.04 mg/ml GMP-grade Liberase MTF (Roche, Mannheim, Germany) for 30 min at 37 °C, and were plated at 5.6–32.0 × 10^4^ cells in a T25 flask (25 cm^2^; Sumitomo Bakelite Co. Ltd., Tokyo, Japan) in Dulbecco’s modified Eagle’s medium (DMEM; Sigma, St. Louis, MO, USA) supplemented with 10% autologous serum (autoserum), 2.5 mg/ml amphotericin B (Bristol-Myers Squibb, Tokyo, Japan), and 0.3% gentamicin (Nitten, Nagoya, Japan) which is only allowed in cell culture for clinical use in Japan and has low cytotoxicity. The scientific rationale for the use of autologous serum is to avoid any potential immune response/reaction to allogeneic and xenogeneic serum. DPSCs were detached by incubation with TrypLE™ Select (Invitrogen) before they attained 70% confluence. Mobilized DPSCs were further isolated by using a stem cell mobilization method under the previously determined optimal conditions: G-CSF (Neutrogin, Chugai Pharmaceutical Co. Ltd., Tokyo, Japan) at final concentration of 100 ng/ml, cell number 2 × 10^4^ cells/100 μl on the Transwell (Corning, Lowell, MA) inserted into 24-well tissue culture plates with an incubation time of 48 h [9]. The isolated MDPSCs were further expanded at 1 × 10^4^ cells/cm^2^ in DMEM (Sigma) supplemented with 10% autologous serum without antibiotics to passage 7 to obtain the required large number of MDPSCs for safety and quality control tests and 10-year cell cryopreservation according to the Japanese guideline of human stem cell clinical research as well as cell transplantation. They were cryopreserved at 1 × 10^6^ cells/ml in a cryoprotectant, CP-1 (Kyokuto Pharmaceutical Industrial Co. Ltd., Tokyo, Japan), by gradually decreasing the temperature to –40 °C at the rate of –2 °C/min and further to –80 °C at the rate of –10 °C/min in a Programed Deep Freezer (Strex, Osaka, Japan). They were stored in a deep freezer (Sanyo Electric Co. Ltd, Osaka, Japan) at –80 °C until use.

### Safety and quality control tests

The final cell product, MDPSCs at passage 7 of culture, was characterized by flow cytometry after immune-labeling with the antigen surface markers CD29, CD44, CD105, and CD31 as described previously [9]. The safety of MDPSCs during the process of tooth transportation, cell processing, cell freezing, and final transplantation was determined by sterility tests for fungi, aerobic and anaerobic bacteria, mycoplasma tests, endotoxin tests, and virus tests. In brief, the MDPSCs at passage 7 after cryopreservation and the MDPSCs combined with collagen and G-CSF used for transplantation in the operating room were sent independently to a quality-control referral laboratory (Tanabe R&D Service Co. Ltd., Saitama, Japan; SRL Inc., Tokyo, Japan; and BML Inc., Tokyo, Japan) for the tests. For the mycoplasma test, the real-time RT-PCR and DNA staining method were used according to the protocol (SRL Inc. and BML, Inc.). The cryopreserved MDPSCs were shipped for transplantation after confirming whether they meet the criteria of MSCs by a battery of in-process quality tests including cell surface marker analysis, cell viability, sterility, endotoxin, mycoplasma, and virus tests.

We examined chromosome aberrations, if any, in cell preparations at passages 9 or 10 of the culture stained with quinacrine mustard and Hoechst 33258 using a standard Q-banding procedure. Karyotypes were analyzed in metaphases of more than 20 cells in accordance with the Human Cytogenetic Nomenclature (ISCN) by entrustment (Chromosome Science Labo Inc., Sapporo, Japan).

### Surgical procedure

**Fig. 1:**
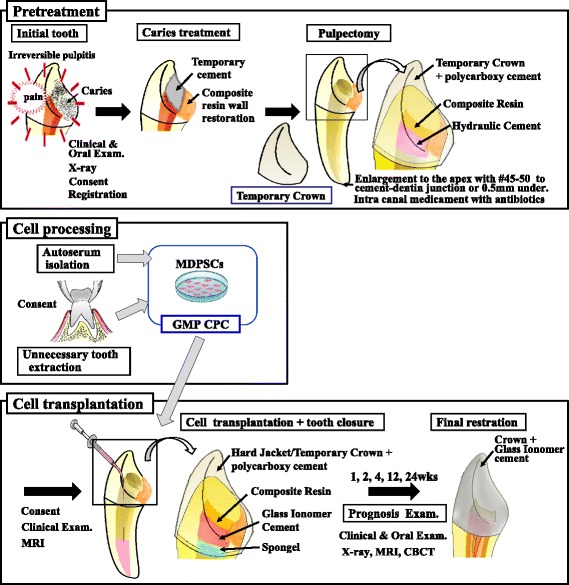
A sequence of illustrations describing step-by-step the sequences of the clinical study, including caries treatment with composite resin wall restoration followed by pulpectomy, cell processing, and cell transplantation, followed by final restoration. *CBCT* cone beam computed tomography, *CPC* Cell Processing Center, *GMP* good manufacturing practice, *MDPSC* mobilized dental pulp stem cell, *MRI* magnetic resonance imaging

Caries of the affected tooth was completely removed. In certain cases it was first necessary to supply a missing wall with composite resin (Clearfil DC core automix, Kuraray Noritake Dental Inc., Tokyo, Japan) with an adhesive procedure using a bonding agent (Clearfil Mega Bond, Kuraray Noritake Dental Inc.) (Fig. 1) to prevent the rubber dam clamp from slipping off the tooth as well as to isolate the root from the saliva and bacteria. The affected tooth was then pulpectomized. Apical shaping was performed to the cemento-dentinal junction or 0.5 mm under from the junction to the size of 0.45 to 0.55 mm after measuring the root canal length with a #25 K file using Root ZX (Morita Corp., Osaka, Japan). After that, the conventional root canal preparation was performed. Irrigation was carried out alternately with 6% NaOCl and 3% H_2_O_2_ and further with saline. An absorbent point moistened with minocycline (MINOMYCIN® IVD, Pfizer Japan Inc., Tokyo, Japan) or 0.5% levofloxacin (CRAVIT®, Santen Pharmaceutical Co. Ltd, Osaka, Japan) was carried into the root canal before cell transplantation as a conventional root canal treatment. The cavity was temporarily filled with a double-seal, water-setting hydraulic cement (Caviton; GC, Tokyo, Japan) and composite resin (Clearfil DC core automix) with an adhesive procedure (Clearfil Mega Bond). Water setting Caviton is advantageous to application of liquid antibiotics in the root canal (Fig. 1). For transplantation, the cryopreserved autologous MDPSCs at 1 × 10^6^ cells were transported to the clean bench of the operating room, thawed, and suspended in 40 μl of a clinical-grade atelocollagen scaffold (Koken, Tokyo, Japan) and 300 ng of G-CSF (Neutrogin) after washing with saline. The root canal was dried well with paper points after irrigation with 3 ml each of 6% NaOCl and 3% H_2_O_2_ and 5 ml of saline, and further with 2 ml of 3% EDTA solution for 2 min (SmearClean, Nippon Shika Yakuhin Co. Ltd., Simonoseki, Japan) and 5 ml of saline. Half of the cell suspension (20 μl) was transplanted into the root canal by a cannula (indwelling needle, #26 gauge, Nipro, Osaka, Japan) paying close attention not to introduce any bubble inside. The Gelatin sponge (Spongel, Astellas Pharma Inc., Tokyo, Japan) was placed on the suspension in the root canal orifice without pressure, and the cavity was sealed with glass ionomer cement (GC Fuji IX EXTRA; GC, Tokyo, Japan) and composite resin (Clearfil DC core automix) with a bonding agent (Clearfil Mega Bond) (Fig. 1). The teeth were further covered with a hard resin jacket crown temporally with polycarboxylate temporary cement (Shofu Hy-Bond temporary cement hard, Shofu) in patients 1 and 3.

### End-points for evaluation and assessment

The patients were followed up at 1, 2, 4, 12, and 24/28/32 weeks after MDPSC transplantation. For the safety evaluation, the incidence, severity, and outcome of immediate or delayed adverse events were recorded. As a first-in-human clinical pilot study under the Japanese guidelines of human stem cell clinical research, urine chemistry examinations and blood tests and blood chemistry examinations were performed at each visit except at 2 weeks. Twelve-lead electrocardiogram was monitored at 4 and 24 weeks. Local clinical examinations including percussion pain and tenderness at each visit and X-ray analyses for periapical lesion were also performed at the first visit (FV), pre-transplantation just before cell transplantation (Pre), and at 4, 12, and 24/28/32 weeks by two radiologists.

Efficacy assessment was performed by the pulp sensibility test using an electric pulp tester (VITALITY SCANNER; Yoshida Dental Trade Distribution Co. Ltd, Tokyo, Japan) at each visit by three dentists. Before electric pulp test (EPT), the surface of the tooth was dried well so as not to flow the current to the adjacent gingival or periodontal tissues. The probe tip was applied to the natural tooth structure, not to the restored part. Toothpaste was used for making good contact with the surface of tooth. The current was slowly increased to give accurate results. Another pulp sensibility test, the cold test, was performed using dicholorofluoromethane refrigerant spray (PULPER, GC Corp., Tokyo, Japan) at every visit. The frozen sponge was applied for a few seconds to the gingival third of the buccal part or any part of the dried tooth to give a good cold conduction. In addition, a 1.5 Tesla (T) MRI (Philips Electronics Japan, Tokyo, Japan) was used for imaging of regenerated tissue at baseline, and at 12 and 24 weeks post-transplantation. Axial fat suppression T2-weighted images (T2WI) were obtained with the use of the Turbo RARE T2 technique. The imaging parameters were: repetition time (TR) 2500 ms, echo time (TE) 70–80, DFOV 22 × 31.6 cm, AQM 336 × 428, average 4, 128 × 128 matrix, 0.234 × 0.234 cm pixel size, 3-mm slice thickness, and 10–20 slices FA 90, NEX 3, EC 1. MRI were analyzed by a computer-assisted manual segmentation (outlining) technique using OsiriX medical imaging software which is a fast DICOM viewer program for the Apple Macintosh (downloadable at www.osirix-viewer.com). The OsiriX program offers all the basic image manipulation functions of zoom, intensity adjustment and filtering with real-time performance. Relative signal intensity (SI) was expressed as the SI of regenerated tissue to SI of the surrounding dentin of the same tooth compared with SI of normal pulp to SI of the surrounding dentin in the opposite site. Relative SI was calculated in axial sections of apical and coronal parts of the root canal, respectively.

Evaluation of dentin formation along the dentinal wall at 16 and 28 weeks was performed by cone beam computed tomography (Alphard-3030, Asahi Roentgen Ind. Co. Ltd., Kyoto, Japan). Cone beam computed tomography images were analyzed using the OsiriX program. At least five measurements were made: the densities of the dental pulp, dentin formation, and dentin were 140–168, 448–525, and 996–1025, respectively. Therefore, the low-density area ranging from 0 to 425 was considered as the dental pulp. The areas with this density range were automatically deducted and the volumes of the dental pulp were calculated.

### Statistical analyses

Data are reported as means ± SD. *P* values were calculated using Student’s *t* test and Tukey’s multiple comparison test method in SPSS 21.0 (IBM, Armonk, NY, USA).

## Results

Five patients with irreversible pulpitis were enrolled from May to December 2013 in this pilot clinical study. Baseline characteristics of each individual patient are depicted in Table 1. Three patients were men and two were women, aged 28.6 ± 10.0 years (range, 20–44 years). Four patients had chronic ulcer pulpitis and one had acute suppurative pulpitis at the time of enrollment. The transplantation of the MDPSCs was performed after 1 to 12 weeks following pulpectomy.

**Table 1:** Baseline characteristics of the individual patients

Characteristic	Patient 1	Patient 2	Patient 3	Patient 4	Patient 5
Age (years)	44	27	20	32	21
Gender	Female	Male	Male	Male	Female
Affected tooth	Upper right 2nd premolar	Lower left first premolar	Upper right first incisor	Lower right 2nd premolar	Upper left first incisor
Caries treatment at the first visit	No treatment	Cement filling with camphorated phenol application	Crown tooth fracture	No treatment	Resin filling
Local clinical findings					
Cold/hot pain	–	–	–/+	–	–
Percussion pain	–	–	–	–	+
Tenderness	–	–	–/+	–	–
Electric pulp test	+ (30)	+ (29)	+ (30)	+ (19)	+ (15)
Pulpitis	Chronic ulcer	Chronic ulcer^a^	Acute suppurative	Chronic ulcer	Chronic ulcer
Unnecessary tooth	Upper right 3rd molar	Upper right 3rd molar	Upper right 3rd molar	Upper left 3rd molar	Upper left 3rd molar
Time between pulpectomy and transplantation	11 weeks	3 weeks	6 weeks	3 weeks	12 weeks

^a^ The positive reaction was detected by electric pulp test (EPT), indicating that the pulp tissue was alive on enrollment. The tooth was left with camphorated phenol application in the cavity for 3 months before root canal treatment, and the pulp tissue became completely necrosis with a periapical lesion at that time

### Outcome of harvest and isolation of MDPSCs

**Fig. 2:**
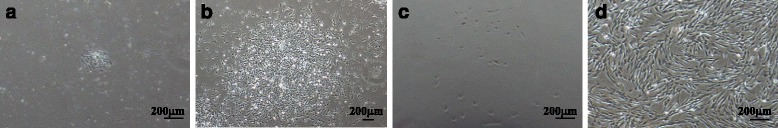
Isolation of MDPSCs from an autologous discarded tooth. **a** Primary DPSCs forming a small colony on day 3. **b** The DPSCs on day 7. The colony increased in size. **c** MDPSCs at passage 2 of culture on day 3. **d** MDPSCs at passage 7 of culture on day 5 before cryopreservation

Human primary DPSCs (Fig. 2a) formed a colony in 7–15 days (Fig. 2b), and clinical-grade human MDPSCs were further isolated utilizing G-CSF-induced stem cell mobilization in the isolator (Fig. 2c). The expanded MDPSCs were stellate with short processes or spindle in shape (Fig. 2d). Flow cytometry revealed that positive rates of CD29, CD44, CD105, and CD31 were 98.7 ± 1.2%, 99.5 ± 0.3%, 94.3 ± 7.9%, and 0.6 ± 0.4%, respectively. The mean total cell number at passage 7 of culture excluding patient 1 was 15.5 ± 4.0 × 10^6^. After the thawing of the frozen cells at passage 7 the cell viability was 83.0 ± 6.7% (Table 2). There were no significant structural chromosomal abnormalities/aberrations in the karyotype of all diploid cells. However, there were a few chromosomal aberrations in patients 1 and 4 (Table 2). In patient 4, 45,X found in one out of 20 cells did not affect regeneration after cell transplantation, possibly due to the fact that the Y chromosome functions only during development. No structural abnormalities including irregular portion of chromosomal DNA and no more than two chromosomes of a pair (trisomy, tetrasomy) were observed. In patient 1, 45,X found in two out of 20 and 45,X,-9 was detected. However, further examination of 45 demonstrated no specific chromosome anomalies. Also, no structural abnormalities and no more than two chromosomes of a pair (trisomy, tetrasomy) were detected. Therefore, cells from patients 1 and 4 could be used safely for cell transplantation. MDPSCs showed no bacterial, fungal, mycoplasma, endotoxin, or virus contamination in the expanded cells at passage 7 of culture after cryopreservation and in the freeze-thawing cells combined with atelocollagen and G-CSF (Table 2).

**Table 2:** Cell biological characteristics, including viability, expression rate of stem cell markers, cell survival rate, and karyotype

Cell characteristics	Patient 1	Patient 2	Patient 3	Patient 4	Patient 5	Average
Total cell product number × 10^6^	1.4^a^	10.3	17.2	19.6	14.8	15.5 ± 4.0^b^
Viability (%)	80.5	93.7	77.3	85.0	78.3	83.0 ± 6.7
Stem cell markers (%)						
CD29	97.0	99.6	97.7	99.6	99.5	98.7 ± 1.2
CD44	99.8	99.4	99.2	99.8	99.1	99.5 ± 0.3
CD105	80.4	99.9	97.0	98.4	95.6	94.3 ± 7.9
CD31	0.9	0.9	0.9	0.0	0.5	0.6 ± 0.4
Karyotype	46,XX (17/20) 45,X (2/20) 45,X,-9 (1/20)	46,XY (20/20)	47,XYY (20/20)	46,XY (19/20) 45,X (1/20)	46,XX (20/20)	

^a^ The mobilized dental pulp stem cells (MDPSCs) of patient 1 were damaged at passage 4 of culture due to the high temperature of the incubator since the air-conditioner was broken in the Cell Processing Center, and the cryopreserved MDPSCs at passage 3 were used for further expansion, resulting in a smaller number of cells at passage 7. Karyotype was analyzed at passage 10

^b^ Excluding patient 1

### Safety evaluation

**Fig. 3:**
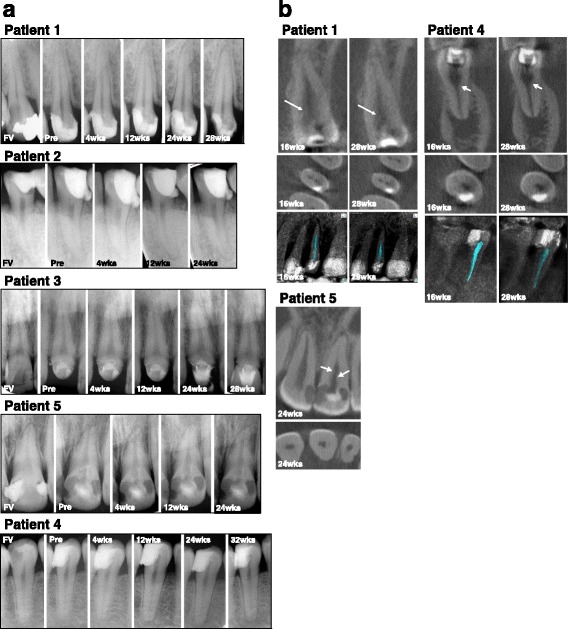
Radiological analyses. **a** X-ray photographic analysis to show the changes and evolution of periapical tissues and apical and/or lateral dentin formation in the root canal at the first visit (*FV*), pre-transplantation just before cell transplantation (*Pre*), and 4, 12, and 24/28 weeks (*wks*) after autologous transplantation of MDPSCs with G-CSF in pulpectomized teeth in five patients. No significant changes were seen in the periapical areas, except in patient 4 who showed widening of periodontal ligament space at 12 weeks and periapical radiolucency at 24 and 32 weeks. Patient 2 preoperatively had periapical radiolucent lesion decreased in area size with a little radiographic periodontal ligament space widening at 24 weeks. **b** Cone beam computed tomography evaluation of apical/lateral dentin formation in the root canal in coronal and axial slices in three patients at 16 and 28/32 weeks. *Arrows* indicate newly formed dentin. The 3D cone beam computed tomography images by the OsiriX program demonstrated a decrease in low-density areas at 28 weeks compared with 16 weeks

No adverse events related to cell transplantation were observed by examination of blood and urine and twelve-lead electrocardiogram during 24 weeks of follow-up in all patients (Table 3). Clinical examinations demonstrated no postoperative pain, including percussion pain and tenderness, at all follow-up visits up to 24 weeks. The radiographic examinations made by two radiologists showed no significant changes in the periapical areas related to the cell therapy in three patients (patients 1, 3, and 5). The periapical lesion clearly diagnosed before transplantation was gradually reduced in size and radiolucency during 24 weeks follow-up. In patient 2 there was minor widening of periodontal ligament space at 24 weeks. There was widening of the periodontal ligament space at 12 weeks and periapical radiolucency at 24 weeks in patient 4 (Fig. 3a).

**Table 3:** Safety tests of mobilized dental pulp stem cells at passage 7 of culture and at cell transplantation

	Patient 1	Patient 2	Patient 3	Patient 4	Patient 5
Bacteria (aerobe, anaerobe, fungus)	–	–	–	–	–
Endotoxin (pg/ml)	<1.0	<1.0	<1.0	<1.0	<1.0
Mycoplasma	–	–	–	–	–
Virus					
Hepatitis B	–	–	–	–	–
HIV-1	–	–	–	–	–
HTLV-1	–	–	–	–	–
Parvovirus	–	–	–	–	–
Hepatitis C	–	–	–	–	–

*HTLV-1* human adult T-cell leukemia virus

### Efficacy evaluation

Assessment of pulp sensibility by EPT was performed in all patients. The EPT demonstrated a negative response before cell transplantation. There was a positive response after 4 weeks in four patients (Table 4), suggesting functional re-innervation in the regenerated pulp tissue. However, patient 2 demonstrated a negative response after 24 weeks of follow-up since there had already been periapical radiolucency at the time of cell transplantation despite a positive response during the enrollment of the patient.

**Table 4:** Safety and efficacy evaluation

Characteristics	Patient 1	Patient 2	Patient 3	Patient 4	Patient 5
Abnormality					
Blood test	–	–	–	–	–
Urine test	–	–	–	–	–
Cardiogram	–	–	–	–	–
Local clinical examination	–	–	–	–	–
Electric pulp test					
Day positive reaction started	+ Day 6 (25, 29, 31) (control right lower 2nd premolar 31)	–^a^	+ Day 6 (55, 58, 69) (control left upper canine 48)	+ Day 28 (49, 64, 80+) (control left lower 2nd premolar 24)	+ Day 14 (45, 62, 80+) (control right upper incisor 13)
12 weeks	+ (34, 38, 36) (control left upper 2nd premolar 45)	–^a^	+ (43, 47, 47) (control left upper incisor 37)	+ (39, 44, 52) (control left lower 2nd premolar 23)	+ (53, 53, 59) (control right upper incisor 11)
24 weeks	+ (40, 41, 44) (control left upper 2nd premolar 41)	–^a^	+ (43, 44, 46) (control left upper canine 25)	+ (50, 57, 57) (control right lower 1st premolar 24)	+ (43, 48, 51) (control right upper incisor 10)
MRI (relative signal intensity of apical part of root canal at 24 weeks)	0.8	0.9	0.9	0.7	0.9
X-ray (periapical radiolucency at 24 weeks)	–	–/+^b, c^	–	+	–
Cone beam computed tomography (lateral dentin formation)	+	–	–	+	+

*MRI* magnetic resonance imaging

^a^ The positive reaction was detected after 36 weeks

^b^ Area of the periapical radiolucency was gradually reduced after cell transplantation

^c^ Widening of periodontal ligament space was detected

**Fig. 4:**
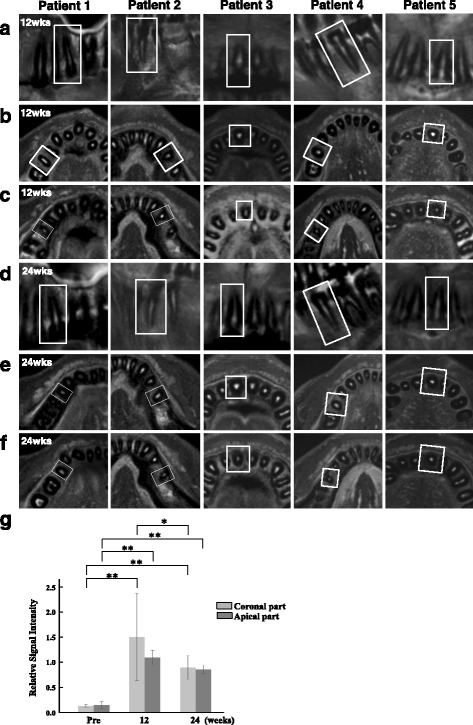
The changes of fat-suppressed T2-weighted (FST2W) MRI in cell-transplanted root canal in five patients. *Squares* indicate the teeth with cell transplantation. **a**–**c** Twelve weeks and **d**–**f** 24 weeks after transplantation of MDPSCs and G-CSF with collagen. **a**,**d** Sagittal slice; **b**,**e** axial slice at the coronal part of the root canal; **c**,**f** axial slice at the apical part of the root canal. **g** The relative SI of MRI. There are significant differences in the relative SI between pulpectomized root canal and cell transplanted root canal at 12 and 24 weeks both in the coronal part and apical part (**P < 0.01); data are expressed as mean ± SD (*n* = 3). There is a significant difference in the relative SI of root canal between 12 weeks and 24 weeks in the coronal part (*P <0.05); data are expressed as mean ± SD (*n* = 4)

Next, the SI in the root canals of the regenerated tissue was examined by MRI. The pulpectomized root canal before cell transplantation served as a negative control, showing low SI in the whole root canal (Fig. 4g). The SI of MRI in the affected teeth demonstrated a gradual decrease after transplantation (Fig. 4g). The SI in the coronal part at 12 weeks was significantly higher compared with that in the coronal part at 24 weeks (*P* < 0.05), suggesting incomplete pulp regeneration in the coronal part at 12 weeks. Evaluation of the SI in the root canal approached that of the normal pulp in untreated controls after 24 weeks. In addition, there was also no significant difference in the SI between the apical and the coronal part of the root canal at 24 weeks, indicating complete pulp regeneration (Fig. 4g).

Dental radiography at 24/28 weeks showed obliteration of the enlarged apical portion following pulpectomy in three cases (patients 1, 3, and 5) (Fig. 3a). The radiographic interpretation on cone beam computed tomography at 28 weeks demonstrated lateral dentin formation in three cases (patients 1, 4, and 5) (Fig. 3b). Further analysis of the low-density area by OsiriX medical imaging software demonstrated that the volumes of the dental pulp at 28 weeks decreased compared with 16 weeks; from 0.0143 cm^3^ to 0.0125 cm^3^ in patient 1 and from 0.0110 cm^3^ to 0.0081 m^3^ in patient 4, respectively. However, in patient 5, the cone beam computed tomography was not obtained at the outset, and therefore the decrease in volume was not determined.

## Discussion

The aim of the present investigation was to assess the safety, potential efficacy, and clinical feasibility of cell-based therapy with autologous MDPSCs and G-CSF for pulp/dentin regeneration in pulpectomized teeth with complete apical closure in patients with irreversible pulpitis. To the best of our knowledge, the present investigation represents the first clinical study of MDPSC transplantation in teeth. Clinical-grade MDPSCs were successfully isolated by utilizing a G-CSF-induced mobilization method in all five patients. Their biological characteristics including expression rate of stem cell markers, total cell number, and cell survival rate were in the normal range, and a sterility test and chromosomal test resulted in no abnormality. A wide variety of clinical trials have assessed the safety of MSC intravascular delivery for graft-versus-host disease, ischemic stroke, Crohn’s disease, myocardial infarction, cardiomyopathy, and so forth, and none of the patients have reported any significant adverse events, including acute infusional toxicity, organ system complication, infection, death, or malignancy associated with the cell therapy [11]. Autologous DPSCs have clinically been transplanted into human mandibles, revealing regeneration of compact bone unlike the usual alveolar spongy bone [12]. There have been no reports, however, in terms of safety on transplantation of DPSCs in any disease in the clinic until now. Our previous preclinical study has demonstrated that MDPSCs isolated from dogs and humans according to GMP conditions when transplanted into NOD/SCID mice or KSN nude mice elicited no tumor formation [9, 10]. These results demonstrated the safety of clinical-grade MDPSCs. Furthermore, canine MDPSCs autologously transplanted in the pulpectomized tooth in dogs demonstrated no tumor formation in any tissues or organs up to 3 months [10]. The present clinical investigation was performed using the protocol used in the canine preclinical study. The results demonstrated no complications related to the transplantation of MDPSCs, consistent with results of other studies on MSCs from a variety of tissues and our canine preclinical study.

The most commonly used methods in clinical practice to determine pulp status are pulp sensibility tests, including the thermal test and EPT [13]. They are not directly related to pulpal vitality, but depend on a subjective response to an external stimulus to the nervous system [13–15]. The EPT can only be used to determine whether or not there is viable tissue in the root canal and cannot be used to determine the degree of pulp disease or vitality [16–18]. Furthermore, no studies have demonstrated any utility of the readings or numerical display [19]. On the other hand, pulp vitality tests to analyze the presence of pulp blood flow by laser Doppler flow or pulse oximetry are considered as better methods of gauging pulp health than sensibility tests [14, 20]. Many practical issues, however, needed to be addressed before the pulp vitality tests become the standard pulp diagnostic test [14]. Pulp sensibility tests supply valuable information, especially when the EPT is used in combination with either CO_2_ snow or refrigerant spray [20]. Thus, we evaluated, at first pulp status, viability by the EPT and cold test. The negative response on EPT before cell transplantation into pulpectomized teeth changed into a positive response after 4 weeks in four cases. This result is comparable with the previous preclinical findings in dogs that pulp tissue is regenerated in 70–80% of the total volume of the root canal with nerve extension to dentin within 4 weeks [10]. It has been suggested that the regenerated tissue could transmit sensory signals by Aδ fibers perceived as pain by electric stimuli [21], which sensory nerves extend from the trigeminal ganglion including nociceptive axons to odontoblasts, as detected in canine regenerated pulp [10]. In this study, the tooth surface was completely dry and is expected to provide reliable data. The shorter the distance between the electrode and the pulp, the lower the resistance to the flow of current becomes [22]. Thus, the numerical value of the electric response in the affected tooth had a high threshold compared with controls since the affected tooth might miss a part of the coronal pulp (Table 4). Canal moisture might be another factor for false-positive response [23]. However, the transition from a negative response before cell transplantation to a positive response following cell transplantation might be considered as evidence of re-innervation. The EPT, however, still has some limitations and shortcomings since it is dependent on subjective perception and description of a response to the electric stimulus by the patient [15]. Therefore, alternative objective diagnosis of the regenerated tissue was further performed by MRI. MRI provides high-resolution images, allowing fine discrimination between blood-filled structures of dental pulp and the adjacent tooth [24, 25]. Soft tissue abnormalities produced by inflammation caused by increased water content are ideally displayed by MRI [26, 27]. MRI has been demonstrated to be a feasible means to visualize changes in the dental pulp, including reperfusion and revitalization of the affected teeth with traumatic dental injury after clinical treatment [25]. Our previous preclinical study demonstrated that the MRI signal intensity (SI) in the regenerated teeth at 24 weeks after cell transplantation was similar to that in normal teeth, and was significantly higher compared to that in control non-regenerated teeth without cell transplantation, suggesting the potential usefulness of MRI to serially assess the regeneration of pulp tissue [28]. In this clinical study, the relative SI of MRI of pulp-like regenerated tissue in both apical and coronal parts at 24 weeks compared with surrounding dentin was similar to that of normal pulp compared with surrounding dentin in four cases. In one case, patient 2, the cavity was applied with camphorated phenol to relieve pain before first visit and was left for more than 3 months after patient enrollment due to the patient’s circumstances, which resulted in apical periodontitis with sinus tract due to coronal leakage. The root canal of the affected tooth was enlarged to 0.25 mm in width to the cemento-dentinal junction and 0.55 mm in width 0.5 mm under from the cemento-dentinal junction and was disinfected with usual root canal treatment twice with intracanal antibiotics prior to cell transplantation. A similar relative SI to other cases was detected in the apical part of the root canal by axial sectional view at 24 weeks. This result may suggest that, even in the case of apical periodontitis, revascularization may occur after cell transplantation, consistent with the findings of a significant decrease in the periapical radiolucent area. Furthermore, in addition to MRI, cone beam computed tomography might be a potential technique to evaluate pulp status. The deposition of tubular/osteodentin along the dentinal wall, referred to as lateral dentin formation, is usually accompanied by pulp regeneration, leading to a reduction of the root canal space as demonstrated by histological analysis [10]. The accurate and highly reproducible calculation of teeth volumes has been reported by a cone beam computed tomography study to estimate the age of adults [29], and to examine the effect of orthodontic treatment [30]. Results in patients 1 and 4 demonstrated that regenerated pulp-like tissue decreased in volume at 28 weeks compared to that at 16 weeks. Thus, quantitative objective evaluation of the volumetric change of regenerated pulp-like tissue following the cell therapy by cone beam computed tomography imaging may be a potent primary end-point.

In patient 4, widening of periodontal ligament space at 12 weeks and periapical radiolucency at 24 weeks was demonstrated by dental radiographical examination. The dental radiograph at 4 weeks, however, demonstrated no change in the periodontal ligament space. The positive response by EPT that started at 4 weeks was not changed at 24 weeks. The low-density area was decreased at 24 weeks compared to that at 12 weeks using the OsiriX program of cone beam computed tomography imaging, indicating lateral dentin formation in the root canal. The dental radiograph at 32 weeks demonstrated no significant increase in periapical radiolucency (Fig. 3a). These results suggest that the transplanted tooth, although once regenerated, might be infected gradually by microleakage [31], especially from the cervical area sealed with composite resin. In addition, one cannot rule out the possibility of prior infection [32]. The anti-inflammatory effect of MDPSCs might inhibit inflammation [9, 10, 33] after cell transplantation for a while; further, long-term follow-up may be necessary to demonstrate pulp/dentin regeneration.

The triad of stem/progenitor cells, a growth factor/migration factor, and scaffold is essential for optimal regenerative endodontics [1]. Our previous preclinical study in dogs demonstrated that MDPSCs are more advantageous than colony-derived DPSCs to regenerate a larger volume of pulp tissue and prevent mineralization inside the root canal [10, 34]. The transplanted MDPSCs did not directly differentiate into endothelial cells, neuronal cells, or pulp cells. Various trophic factors secreted by MDPSCs could enhance migration and proliferation of endogenous stem/progenitor cells from the surrounding tissues. MDPSCs could also regulate inflammation with immunosuppressive and immunomodulatory properties [10]. Thus, in the present study, MDPSCs were used to enhance pulp regeneration. On the other hand, G-CSF was used as a growth/migration factor for this clinical study, since G-CSF has been approved by the Pharmaceuticals and Medical Devices Agency, Japan (PMDA), the US Food and Drug Administration (FDA), and the European Medicines Agency (EMA). G-CSF is available as a drug product for treatment of neutropenia and for reconstitution of bone marrow to mobilize hematopoietic stem cells from bone marrow [35, 36], with only a few well-described side effects. In clinics, G-CSF treatment resulted in a positive functional effect in stroke [37–39]. Furthermore, the combined local application therapy of G-CSF with MSCs has demonstrated augmented spinal cord regeneration [40], peripheral nerve regeneration [41], cerebral ischemia recovery [42], ulcerative colitis improvement [43], and myocardial infarction recovery [44] in experimental animal models. A case report has recently demonstrated neurological improvement of spinal cord injury using the combination therapy of G-CSF and autologous bone marrow stem cells [45]. Our previous preclinical study has demonstrated that G-CSF reduces apoptosis of the transplanted MDPSCs and localizes the transplanted cells in the root canal. Transplantation of MDPSCs together with G-CSF yielded a significant larger volume of regenerated pulp tissue compared with transplantation of G-CSF alone or MDPSCs alone. Neurite outgrowth was also significantly increased and inflammation was significantly reduced in the transplants of MDPSCs and G-CSF together compared with either alone [10]. In the present clinical study, combinatorial effects of G-CSF with MDPSCs may be consistent with the previous preclinical findings, suggesting it as a promising therapeutic regulator of MSCs that can improve therapeutic outcomes.

The ultimate goal for pulp/dentin regeneration is functional recovery of teeth to prolong their life. The present study demonstrated a positive reaction in the EPT and similar SI of MRI in the root canal to normal pulp, indicating that regenerated tissue can transmit sensory signals and recover vascular supply. Obliteration of the enlarged apex and lateral dentin formation in the pulpectomized tooth were advantageous to prevent tooth fracture, although excessive dentin formation like pulp stones in the center of regenerated tissue may lead to less vascularization of the tooth and to fragility. The possible factors to induce higher mineralization in the regenerated tissue, including transplanted cell types, scaffold, and microenvironment, need to be further elucidated to prevent excessive dentin formation in the root canal. Furthermore, the re-innervation has critical roles in pulp homeostasis and defense mechanisms including blood flow [46], extravasation of immune and inflammatory cells [47, 48], and dentin regeneration [1, 49].

One critical hurdle that still needs to be overcome to enable more comprehensive clinical adoption is infection control during root canal treatment by root canal irrigants and intracanal medicaments, and after cell transplantation by the antimicrobial scaffold. A widely used medicament, calcium hydroxide paste, might inhibit good pulp regeneration if it remained in the root canal. Another critical challenge to overcome is dentin formation to completely and rapidly cover the regenerated pulp, thus preventing microleakage.

## Conclusion

In this pilot clinical study, the safety of MDPSC transplantation in pulpectomized teeth was demonstrated. The efficacy of the combinatorial regenerative therapy of MDPSCs with G-CSF for pulp/dentin regeneration was also suggested by EPT, MRI, and cone beam computed tomography. Further randomized clinical trials with a large number of patients is warranted before regenerative endodontics based on mobilized dental pulp stem cells will become a reality.

## Abbreviations

DPSC: Dental pulp stem cell
EPT: Electric pulp test
G-CSF: Granulocyte colony-stimulating factor
GMP: Good manufacturing practice
MDPSC: Mobilized dental pulp stem cell
MRI: Magnetic resonance imaging
MSC: Mesenchymal stem cell
SI: Signal intensity
